# Transcriptomic Response of the Atlantic Surfclam (*Spisula solidissima*) to Acute Heat Stress

**DOI:** 10.1007/s10126-024-10285-0

**Published:** 2024-01-19

**Authors:** Michael Acquafredda, Ximing Guo, Daphne Munroe

**Affiliations:** Haskin Shellfish Research Laboratory, Department of Marine and Coastal Sciences, Rutgers University, 6959 Miller Avenue, Port Norris, NJ 08349 USA

**Keywords:** Atlantic surfclam, *Spisula solidissima*, Selective breeding, Transcriptome, Heat stress, Aquaculture

## Abstract

There is clear evidence that the oceans are warming due to anthropogenic climate change, and the northeastern coast of USA contains some of the fastest warming areas. This warming is projected to continue with serious biological and social ramifications for fisheries and aquaculture. One species particularly vulnerable to warming is the Atlantic surfclam (*Spisula solidissima*). The surfclam is a critically important species, linking marine food webs and supporting a productive, lucrative, and sustainable fishery. The surfclam is also emerging as an attractive candidate for aquaculture diversification, but the warming of shallow coastal farms threatens the expansion of surfclam aquaculture. Little is known about the adaptive potential of surfclams to cope with ocean warming. In this study, the surfclam transcriptome under heat stress was examined. Two groups of surfclams were subjected to heat stress to assess how artificial selection may alter gene expression. One group of clams had been selected for greater heat tolerance (HS) and the other was composed of random control clams (RC). After a 6-h exposure to 16 or 29 °C, gill transcriptome expression profiles of the four temperature/group combinations were determined by RNA sequencing and compared. When surfclams experienced heat stress, they exhibited upregulation of heat shock proteins (HSPs), inhibitors of apoptosis (IAPs), and other stress-response related genes. RC clams differentially expressed 1.7 times more genes than HS clams, yet HS clams had a stronger response of key stress response genes, including HSPs, IAPs, and genes involved with mitigating oxidative stress. The findings imply that the HS clams have a more effective response to heat stress after undergoing the initial selection event due to genetic differences created by the selection, epigenetic memory of the first heat shock, or both. This work provides insights into how surfclams adapt to heat stress and should inform future breeding programs that attempt to breed surfclam for greater heat tolerance, and ultimately bring greater resiliency to shellfish farms.

**Supplementary Information:**

The online version contains supplementary material available at 10.1007/s10126-024-10285-0.

## Introduction

There is clear evidence that the oceans are warming due to anthropogenic climate change (Intergovernmental Panel on Climate Change [IPCC] 2014; Ishii et al. 2017; Resplandy et al. 2018; Chan et al. 2019; Zanna et al. 2019). The northeastern coast of the USA contains some of the fastest warming areas in the world and has experienced episodic marine heat waves (Pershing et al. 2015; Saba et al. 2016; Northeast Fisheries Science Center [NEFSC] 2020). Since 2000, this region has also undergone a regime shift, experiencing a significant increase in the number of warm core rings, which could bring warmer, saltier water from the Gulf Stream to the continental shelf (Gangopadhyay et al. 2019). Ocean warming is projected to cause serious biological and social implications for fisheries and aquaculture, and this region has already experienced numerous socioecological changes driven or exacerbated by this phenomenon. This includes the collapse of the Gulf of Maine cod (*Gadus morhua*) fishery (Pershing et al. 2015), shifting spatial distributions of numerous finfish species (Nye et al. 2009; Kleisner et al. 2017; Free et al. 2019; Morson et al. 2019), and heat-related mortalities in blue mussel (*Mytilus edulis*) aquaculture (Mallet et al. 1990; LeBlanc et al. 2005).

One species particularly vulnerable to ocean warming is the Atlantic surfclam (*Spisula solidissima*), because high temperatures reduce its growth and increase its mortality (Goldberg and Walker 1990; Weinberg 2005; Acquafredda et al. 2019, 2020). The surfclam is one of the largest non-symbiotic suspension feeding bivalves, and it plays an ecologically important role linking primary productivity to higher trophic-level consumers in the nearshore ecosystem (Munroe et al. 2013). The surfclam is a principal prey item for many species, including the horseshoe crab (*Limulus polyphemus*; Botton and Haskin 1984), the moon snail (*Euspira heros*), the lady crab (*Ovalipes ocellatus*), the rock crab (*Cancer irroratus*; Mackenzie et al. 1985), haddock (*Melanogrammus aeglefinus*), and cod (Clark 1954). In the USA, the surfclam also supports a productive, lucrative, and sustainable fishery. As of 2017, the fleet landed 40.2 million pounds of meats valued at 32.7 million USD, and the fishery was neither overfished nor was overfishing occurring (NEFSC 2017; National Marine Fisheries Service [NMFS] 2018). However, across its range, fishable stocks of surfclams have shifted away from southern and shallow locations towards more northern and deeper areas (Munroe et al. 2013, 2016; Powell et al. 2016; Hennen et al. 2018; Hofmann et al. 2018; Timbs et al. 2019). The surfclam is also emerging as an attractive aquaculture species in the region since it is native, grows rapidly, and complements the growing seasons of other regionally farmed bivalves (Acquafredda et al. 2019; Acquafredda and Munroe 2020; Acquafredda et al. in review). Warming of shallow coastal farms consequently threatens the expansion of surfclam aquaculture.

The adaptive potential of the surfclam to cope with heat stress is an active area of research. Juvenile surfclams that survived prolonged heat stress had greater survival upon re-exposure to those conditions as adults compared to clams that had never experienced severe heat stress (Acquafredda et al. 2020). Moreover, first generation progeny of heat-selected surfclams survived significantly longer during a lethal heat challenge compared to control progeny bred from non-selected individuals (Acquafredda et al. 2020). Together, these findings suggest that heat tolerance is a heritable trait in surfclams, and selective breeding may produce surfclams with greater heat tolerance for farmers seeking to cultivate this species.

Heat stress causes oxidative damage (Verlecar et al. 2007), immune system deficiencies (Chen et al. 2017), impairment of feeding processes, and energy budget reductions (Ezgeta-Balić et al. 2011) in marine bivalves. Research has shown that in surfclams specifically, heat stress decreases clearance rate, respiration rate, and assimilation rate, thereby reducing scope for growth and negatively impacting survival (Hornstein et al. 2018; Acquafredda et al. 2019). Although the phenotypic response of surfclams to heat stress has been documented, little is known about the molecular underpinnings of the surfclam’s response to heat stress.

While only a few species have been studied, some patterns have emerged across the gene expression profiles of bivalves experiencing heat stress. The Pacific oyster (*Crassostrea gigas*) upregulates genes associated with stress response (e.g., heat shock proteins), lipid biosynthesis, and immune response during exposure to unfavorably high temperatures (Meistertzheim et al. 2007; Lang et al. 2009). It also suppresses growth and downregulates genes that encode for lipid catabolism and mobilization (Meistertzheim et al. 2007; Lang et al. 2009). Manila clams (*Ruditapes philippinarum*) are known to increase expression of stress and immune response genes when subjected to heat stress (Menike et al. 2014; Ding et al. 2018). Multiple studies also suggest that genes that mitigate the effects of reactive oxygen species (ROS) are upregulated during thermal stress (Meistertzheim et al. 2007; Truebano et al. 2010; Menike et al. 2014; Song et al. 2022). In addition to the upregulation of HSPs and antioxidant genes such as SODs, inhibitors of apoptosis, an expanded gene family in most bivalves, are also upregulated by heat and other stressors (Zhang et al. 2012a, b; Guo et al. 2015; Song et al. 2021).

In this study, the surfclam transcriptome under heat stress was examined. Two groups of clams were used, one of which had been selected for greater heat tolerance via an acute heat stress 4 months prior to the study, and the other is a group of randomly selected control clams that never experienced severe heat stress. After a 6-h exposure to 16 or 29 °C, gill transcriptome expression profiles of the four temperature × group combinations were obtained and compared. Specifically, genes and pathways that were differentially expressed by these surfclam groups were identified and analyzed, providing insight into molecular mechanisms of heat response and tolerance.

## Materials and Methods

### Experimental Design

A cohort of 21-month-old Atlantic surfclams (*Spisula solidissima*) originally spawned in 2017 and farm-raised in Barnegat Bay, NJ, were exposed to control conditions (~12 °C) or a lethal heat challenge (~ 28 °C) for a selective breeding study (Acquafredda et al. 2020). Over the 5-day challenge, mortality reached ~ 55%. However, latent mortality occurred for an additional month, leading to a final mortality of ~ 75% (Acquafredda et al. 2020). In that study, the heat-selected clams were designated Heat-Selected-17, or HS-17; in the present study, this group will simply be referred to as HS. Likewise, the non-selected clams (randomly chosen control individuals) used in that study were designated Non-Selected-17, NS-17, but in the present study, this group will be referred to as Random-Control, or RC.

Four months following the challenge, a controlled experiment was conducted to examine the gene expression patterns of surfclams under favorable and stressful high temperature conditions, and to compare the patterns between clams that previously survived a lethal heat stress to those that never experienced such conditions. The experiment was conducted at the New Jersey Aquaculture Innovation Center (AIC) at Rutgers University in North Cape May, NJ. Eighteen individuals were randomly selected from each of the two aforementioned groups, HS and RC. Nine from each group were exposed to a 6-h heat shock (29.0 ± 0.1 °C) and were designated either RC29 or HS29. Likewise, the other nine from each group spent 6 h in favorable control conditions (16.0 ± 0.5 °C) and were designated either RC16 or HS16. No acclimation period was afforded to the individuals that were placed in the heat shock conditions. Instead, clams were moved out of 15.8 °C water and immediately placed into the experimental conditions. Within each treatment, the nine clams from each group were divided into three replicate buckets, each containing three clams and 15 L of treated (1 µm filtered, UV-sterilized) seawater. Buckets in the heat shock treatment shared a common water bath, which was heated with multiple aquarium heaters (300–400 W Aqueon) controlled by dual-stage digital temperature controllers (Inkbird ITC-308). Buckets in the control treatment also shared a common water bath, and its temperature was maintained with an immersion chiller (Aqua Logic, Cyclone 1/4 HP CY-3) and a single-stage digital temperature controller (Aqua Logic, Inc.). A YSI model 86 was used to collect temperature and salinity data, the latter of which ranged from 30.5 to 31.3 across all experimental units. All buckets were continuously aerated and dissolved oxygen concentration data were collected with a YSI model 55. Dissolved oxygen ranged from 5.90 to 6.65 mg L^−1^ in the heat shock treatment and from 8.48 to 9.67 mg L^−1^ in the control treatment. The mean shell length for the HS and RC clams were statistically similar at 35.47 ± 1.34 and 35.25 ± 1.94 mm, respectively (two-sample *t*-test, *p* = 0.70).

After the 6-h experiment, 12 pooled samples (2 clam groups × 2 temperature treatments × 3 replicate buckets) were collected; each was composed of gill tissue from the three clams in each replicate bucket. Gill tissue was selected for sampling because it has been shown to respond to thermal stress in other marine bivalves (Meistertzheim et al. 2007; Lang et al. 2009; Ding et al. 2018; Song et al. 2022). Each clam was shucked with a sterilized scalpel and gill tissue was dissected using sterilized forceps. The tissue of each pooled sample was stored in a 1.5-mL Eppendorf tube filled with RNAlater. The RNAlater in each tube was replaced 24 h after sampling. The samples were then sent to Novogene (CA, USA) for RNA extraction, library construction, and sequencing. After the RNA was extracted from gill tissues and before libraries were constructed, extensive quality control measures were conducted. RNA degradation and contamination were assessed using agarose gel (1%) electrophoresis. Preliminary RNA quantification and purity were assessed with a Nanodrop spectrophotometer (Thermo Fisher, USA). The RNA quality was assessed by determining the RNA integrity number for each sample by using an Agilent Bioanalyzer 2100 (Agilent Technologies, USA). After the quality control procedures, the NEBNext® Ultra™ RNA Library Prep Kit (New England Biolabs, Inc., USA) was used to prepare the samples for sequencing. The mRNA was enriched from total RNA using oligo(dT) beads, and the mRNA was then fragmented randomly in fragmentation buffer. First-strand cDNA synthesis was carried out with random hexamers and reverse transcriptase. A custom second-strand synthesis buffer (Illumina) was added with dNTPs, RNase H and *Escherichia coli* polymerase I to generate the second strand by nick translation. The final cDNA libraries were ready following purification with AMPure XP beads, terminal repair, A-tailing, ligation of sequencing adapters, size selection, and PCR enrichment. Library concentration was first quantified using a Qubit 2.0 fluorometer (Life Technologies, USA). Libraries were then diluted to 1 ng μL^−1^ before checking insert size on an Agilent Bioanalyzer 2100 (Agilent Technologies, USA), and then quantified to greater accuracy using quantitative PCR (Q-PCR) (library activity > 2 nM). High-quality cDNA libraries were then sequenced by an Illumina HiSeq platform. Clean reads were obtained from the raw reads by removing low-quality sequences, sequences with adaptor contamination, and sequences with uncertain nucleotides constituting more than 10% of the read (*N* > 10%).

### Transcriptome Analyses

Trinity (Grabherr et al. 2011) was used to assemble a reference transcriptome since the surfclam genome has not been sequenced. Corset (Davidson and Oshlack 2014), which clusters contigs based on shared reads and separates contigs when different expression patterns between samples are observed, was used for the hierarchical clustering. Longest transcripts of each cluster were designated as unigenes. Seven databases including National Center for Biotechnology Information (NCBI) non-redundant protein sequences, NCBI nucleotide sequences, NCBI euKaryotic Orthologous Groups (KOG), Protein family (Pfam), Swiss-Prot (UniProt Knowledge Base consortium), Gene Ontology (GO), and Kyoto Encyclopedia of Genes and Genome (KEGG) were used to annotate the resulting transcripts and identify genes (or homologs of genes) based on sequence homology. RSEM (Li et al. 2011) was used to convert read counts to fragments per kilobase of transcript sequence per millions base pairs sequenced (FPKM) and was used to determine expression levels. Subsequently, a hierarchical clustering analysis was used to identify differences and patterns of gene expression across the group/temperature comparisons. The clustering analysis was applied to the union of, or common set, of differentially expressed genes (DEGs) found across all pairwise group/temperature comparisons. A complete list of all DEGs with associated information from all seven databases can be found in the Supplemental Information. KEGG pathway analysis was conducted to identify significantly enriched metabolic or signal transduction pathways; this analysis compared the frequency of DEGs to the background frequency of genes associated with a given pathway. Raw sequence data were submitted to the National Center for Biotechnology Information (NCBI) Sequence Read Archive (SRA) under BioProject ID #PRJNA596792 (biosample numbers: SAMN13638038–SAMN13638049); the Transcriptome Shotgun Assembly (TSA) project has been deposited at DDBJ/EMBL/GenBank under the accession number: GKQA00000000.

### Statistical Analyses

**Fig. 1:**
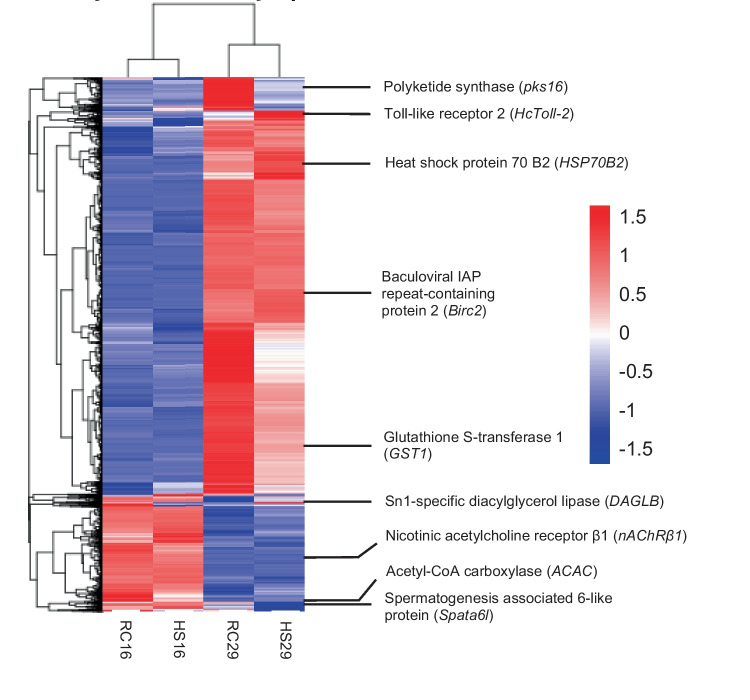
FPKM cluster analysis of differentially expression genes. Clustered using the log_10_(FPKM + 1) values. Red denotes genes with high expression levels, and blue denotes genes with low expression levels

All measures of dispersion reported in this paper are standard deviation, unless otherwise noted. DESeq was the software package used for the differential gene expression analysis (Anders and Huber 2010). The resulting *p* values from the DESeq analysis were corrected using the Benjamini and Hochberg’s approach for controlling the false discovery rate (FDR). Genes with an adjusted *p* value < 0.05 were designated as DEGs. A built-in R package (version 1.1.383–© 2009–2017 RStudio, Inc.) called pheatmap was used to conduct the clustering analysis of DEGs and to generate the hierarchical heat map. In the heat map, DEGs were clustered based on the centered and normalized log_10_(FKPM + 1) values (Fig. 1). For a given group/temperature comparison, the fold change in expression of each mentioned gene group (e.g., heat shock proteins, inhibitors of apoptosis) was calculated as the mean read count of all DEGs identified by the aforementioned databases as being a member of that group or family. For the KEGG pathway analysis, significance was assessed using Fisher’s exact test (hypergeometric test) with the Benjamini and Hochberg’s FDR correction. Any pathway with an adjusted *p* value < 0.05 was designated as significantly enriched.

## Results

### Transcriptome Reconstruction

A total of 555,039,028 raw reads were obtained from the 12 pooled samples containing tissue from 36 clams. After quality control, 541,307,436 clean reads, or 97.53% of all raw reads, were acquired. The mean GC content of sequencing reads across all samples was 37.84 ± 0.38%. The mean percentage of total reads mapped to the reference transcriptome assembly was 72.60 ± 1.32%, and 43.05% were annotated in at least one of seven functional gene databases.

A total of 185,825 transcripts were identified after de novo assembly of the sample transcriptomes, and of those, 185,675 were classified as unigenes. The mean length of the transcripts was 1142 nucleotides, while the median was 692. The minimum and maximum lengths were 201 and 29,827 nucleotides, respectively. This descriptive information was identical for the identified unigenes, with the exception that the mean length of the unigenes was 1143 nucleotides. Overall, 6086 genes were used in the cluster analysis, which depicts differential gene expression across group and temperature. Noteworthy and highly significant DEGs with their Swiss-Prot IDs and descriptions are assembled in Tables 1, 2, and 3. The complete list of DEGs can be found in the Supplemental Information.

**Table 1:** Most notable differentially expressed genes (DEGs) induced by heat stress. HS29 = Heat-Selected clams at 29 °C; HS16 = Heat-Selected clams at 16 °C; RC29 = Random-Control clams at 29 °C; RC16 = Random-Control clams at 16 °C. Unless otherwise noted (*), the Transcript ID for each DEG listed in this table contains the prefix “Cluster-17909.” Please see the Supplemental Information for additional information and complete list of DEGs

ID	Read count HS29	Read count HS16	Fold change (log2[HS29/HS16])	Adjusted *p* value HS29vs.HS16	Read count RC29	Read count RC16	Fold change (log2[RC29/RC16])	Adjusted *p* value RC29vs.RC16	Swissprot ID	Swissprot description
73258	709	74	3.27	5.73E − 04	797	68	3.54	9.14E − 06	Q64433	10-kDa heat shock protein, mitochondria, *Mus musculus*
113554	11,343	16	9.50	6.12E − 19	7062	52	7.10	1.46E − 10	P02518	Heat shock protein 27, *Drosophila melanogaster*
21519	26,739	0	15.74	3.93E − 39	11,694	0	15.08	1.91E − 04	P41825	Heat shock protein 70 A1, *Anopheles albimanus*
21840	23,330	0	16.33	9.97E − 40	15,733	1	14.02	2.19E − 43	P41827	Heat shock protein 70 B2, *A. albimanus*
73231	375,787	13	14.82	1.09E − 21	118,870	14	13.06	1.06E − 12	P41827	Heat shock protein 70 B2, *A. albimanus*
107948	2121	1	11.72	3.06E − 10					P34933	Heat shock-related 70-kDa protein 2, *Bos taurus*
79243	195,584	10,219	4.26	2.96E − 04	175,553	9155	4.26	7.58E − 06	O02192	Heat shock protein 83, *Drosophila auraria*
83430	148,425	7046	4.40	6.58E − 05	133,078	6912	4.27	2.32E − 06	P34058	Heat shock protein HSP 90-beta, *Rattus norvegicus*
64477	14,808	49	8.25	5.29E − 21	9337	45	7.70	4.78E − 05	Q94738	97-kDa heat shock protein, *Strongylocentrotus franciscanus*
71067	42,312	199	7.73	5.41E − 17	38,474	213	7.50	3.94E − 10	Q92598	Heat shock protein 105 kDa, *Homo sapiens*
45742	129,362	274	8.89	1.67E − 15	58,985	335	7.46	5.64E − 04	Q5BIP8	DnaJ homolog subfamily B member 5, *B. taurus*
123126	28,000	49	9.16	2.46E − 22	16,800	68	7.95	1.59E − 24	Q99P31	Hsp70-binding protein 1, *M. musculus*
123681	26	0	6.90	1.01E − 02					Q8BK64	Activator of 90-kDa heat shock protein ATPase homolog 1, *M. musculus*
44006					6987	96	6.18	3.03E − 17	O95433	Activator of 90-kDa heat shock protein ATPase homolog 1, *H. sapiens*
79018	1579	197	3.00	7.28E − 04	2287	217	3.40	3.87E − 06	P27824	Calnexin, *H. sapiens*
61784	15,474	49	8.30	2.23E − 18	9649	53	7.52	4.25E − 23	Q62210	Baculoviral IAP repeat-containing protein 2, *M. musculus*
82696	9741	111	6.46	3.61E − 15	6556	157	5.38	6.30E − 10	Q24307	Death-associated inhibitor of apoptosis 2, *D. melanogaster*
107780	123	2	6.14	7.42E − 04	88	5	4.18	1.49E − 03	Q60989	E3 ubiquitin-protein ligase XIAP, *M. musculus*
128226	437	0	Inf	3.71E − 07	284	0	Inf	8.49E − 03	Q6R7D0	Putative apoptosis inhibitor ORF99, Ostreid herpesvirus 1 (isolate France)
82358	747	151	2.31	4.51E − 02					P41436	Apoptosis inhibitor IAP, Cydia pomonella granulosis virus (isolate Mexico/1963)
64488	1293	283	2.19	4.12E − 02					Q13625	Apoptosis-stimulating of p53 protein 2, *H. sapiens*
62315	323	2	7.35	2.95E − 02					Q16611	Bcl-2 homologous antagonist/killer, *H. sapiens*
62312					208	1	7.68	2.57E − 02	Q16611	Bcl-2 homologous antagonist/killer, *H. sapiens*
45629	41	0	Inf	2.48E − 02					Q8MJC3	Caspase-3, *Oryctolagus cuniculus*
91728					1000	209	2.26	8.85E − 03	Q08DY9	Caspase-3, *B. taurus*
115179	47	2	4.41	1.71E − 02					O08727	Tumor necrosis factor receptor superfamily member 11B, *R. norvegicus*
17060					43	0	Inf	4.41E − 05	O08712	Tumor necrosis factor receptor superfamily member 11B, *M. musculus*
80224	11,325	2285	2.31	2.40E − 02					Q8NFZ5	TNFAIP3-interacting protein 2, *H. sapiens*
80223					3157	670	2.24	6.17E − 03	Q8NFZ5	TNFAIP3-interacting protein 2, *H. sapiens*
40442	124	0	Inf	5.87E − 10	58	2	4.83	7.59E − 04	F7D3V9	leucine-rich repeat-containing G-protein coupled receptor 5, *Xenopus tropicalis* (GenBank description: *HcToll-2*, *Hyriopsis cumingii*, AIA66467.1)
91087	243	0	Inf	2.59E − 03					Q9MYW3	Toll-like receptor 4, *Equus caballus*
62923	211	16	3.72	9.29E − 04					Q6R5N8	Toll-like receptor 13, *M. musculus*
76880	32	198	−2.63	4.73E − 02	17	216	−3.63	2.91E − 04	F6R2G2	NLR family CARD domain-containing protein 4, *X. tropicalis*
52727	369	47	2.97	5.93E − 03						Uncharacterized by Swissprot (GenBank: predicted NLRC5-like, *Strongylocentrotus purpuratus*, XP_011660592.1)
101224					160	655	−2.03	2.79E − 02	Q9N2M8	Headcase protein, *D. melanogaster*
82703	56	333	−2.57	2.54E − 02	90	383	−2.08	4.53E − 02	P47845	Galectin-3, *O. cuniculus*
74838	898	6	7.18	8.38E − 15	868	0	Inf	8.32E − 07	A3FM55	C-type lectin 1, *Hydrophis hardwickii*
75244					189	3259	−4.11	1.47E − 02	Q67EQ1	C-type lectin domain family 4 member E, *R. norvegicus*
113304	16	0	Inf	2.59E − 02					A8QMS7	Myeloid differentiation primary response protein MyD88, *Takifugu rubripes*
Cluster-1827.3	49	0	7.42	6.10E − 03	43	0	6.45	5.98E − 04	Q8HXQ1	Superoxide dismutase [Cu–Zn], *Macaca fascicularis*
57252	96	0	Inf	2.18E − 08	219	0	Inf	4.48E − 04	P46436	Glutathione S-transferase 1, *Ascaris suum*
114824	5685	49	6.85	3.48E − 04					Q3UQ28	Peroxidasin homolog, *M. musculus*
13852					208	6	5.13	1.94E − 07	Q1ENI8	Peroxidasin homolog, *Caenorhabditis elegans*
43138	1487	259	2.52	1.00E − 02					Q8CDN6	Thioredoxin-like protein 1, *M. musculus*
124151	61	0	Inf	2.93E − 06	72	2	5.43	1.66E − 04	Q9UHD2	Serine/threonine-protein kinase TBK1, *H. sapiens*
120157	830	67	3.63	5.46E − 05	1127	59	4.26	4.30E − 08	P47809	Dual specificity mitogen-activated protein kinase kinase 4, *M. musculus*
95615					0	89	−Inf	2.88E − 07	Q5BIS9	5′-AMP-activated protein kinase subunit beta-1, *B. taurus*
76792					11,824	3467	1.77	3.84E − 02	Q04861	Nuclear factor NF-kappa-B p105 subunit, *Gallus gallus*
69500	12,802	410	4.97	9.07E − 03	9721	403	4.59	3.43E − 11	Q03017	NF-kappa-B inhibitor cactus, *D. melanogaster*
16418	69	0	7.28	1.86E − 05					Q9R0T8	Inhibitor of nuclear factor kappa-B kinase subunit epsilon, *M. musculus*
92590	4	84	−4.48	1.08E − 03					Q6AYJ3	Spermatogenesis associated 6-like protein, *R. norvegicus*
92585					19	130	−2.78	1.84E − 02	Q6AYJ3	Spermatogenesis associated 6-like protein, *R. norvegicus*
77318					30	409	−3.76	1.93E − 05	Q86SH2	Zygote arrest protein 1, *H. sapiens*
47189	0	31	−Inf	5.68E − 03					O60290	Zinc finger protein 862, *H. sapiens*
30929					2	39	−4.27	2.28E − 02	P17035	Zinc finger protein 28, *H. sapiens*
72423	0	40	−Inf	1.43E − 03	7	77	−3.51	1.94E − 02	Q9Y6I7	WD repeat and SOCS box-containing protein 1, *H. sapiens*
38828	17	164	−3.28	3.27E − 03	16	161	−3.34	7.24E − 03	Q56R14	E3 ubiquitin-protein ligase trim33, *Xenopus laevis*
70385	7.3	132	−4.17	1.35E − 03	6	97	−4.11	3.71E − 03	P04755	Acetylcholine receptor subunit beta-like 1 (nicotinic acetylcholine receptor β1), *D. melanogaster*

**Table 2:** Most notable differentially expressed genes (DEGs), comparing HS29 and RC29. HS29 = Heat-Selected clams at 29 °C; RC29 = Random-Control clams at 29 °C. The Transcript ID for all DEGs listed in this table contain the prefix “Cluster-17909.” Please see the Supplemental Information for additional information and complete list of DEGs

Transcript ID	Read count (HS29)	Read count (RC29)	Fold change (log2[HS29/RC2])	Adjusted *p* value	Swissprot ID	Swissprot description
118366	0	164	−Inf	1.01E − 08	Q869W9	Probable polyketide synthase 16, *Dictyostelium discoideum*
84331	6	455	−6.21	1.35E − 07	P18320	Profilin, *Heliocidaris crassispina*
88863	0	99	−Inf	1.56E − 05	A0JPI9	Leucine-rich repeat-containing protein 74A, *Rattus norvegicus*
67247	0	72	−Inf	6.69E − 05	P11029	Acetyl-CoA carboxylase, *Gallus gallus*
22731	0	48	−Inf	2.17E − 03	O96006	Zinc finger BED domain-containing protein 1, *Homo sapiens*
117326	0	64	−Inf	2.00E − 02	A4IHD2	Helicase ARIP4, *Xenopus tropicalis*
111156	0	36	−Inf	2.52E − 02	A0JPH4	Sterol regulatory element-binding protein cleavage-activating protein, *X. laevis*
118676	0	33	−Inf	2.80E − 02	Q0VC00	Phospholipase ABHD3, *Bos taurus*
39781	0	33	−Inf	2.80E − 02	Q5S007	Leucine-rich repeat serine/threonine-protein kinase 2, *H. sapiens*
96023	0	32	−Inf	3.50E − 02	Q8WXG6	MAP kinase-activating death domain protein, *H. sapiens*
123853	0	32	−Inf	3.55E − 02	O08727	Tumor necrosis factor receptor superfamily member 11B, *R. norvegicus*
79448	1093	64	4.10	5.73E − 06	Q96SJ8	Tetraspanin-18, *H. sapiens*
113190	57	0	Inf	1.75E − 04	Q9JM53	Apoptosis-inducing factor 1, mitochondrial, *R. norvegicus*
94775	57	0	Inf	2.10E − 04	O08874	Serine/threonine-protein kinase N2, *R. norvegicus*
116707	42	0	Inf	2.17E − 03	F7D3V9	Leucine-rich repeat-containing G-protein coupled receptor 5, *X. tropicalis* (GenBank description: *HcToll-2*, *Hyriopsis cumingii*, AIA66467.1)
112715	347	0	Inf	2.46E − 02	Q8NCG7	Sn1-specific diacylglycerol lipase beta, *H. sapiens*
102707	30	0	Inf	2.47E − 02	Q8BGC0	HIV Tat-specific factor 1 homolog, *M. musculus*
103534	39	0	Inf	3.51E − 02	P23906	Interferon regulatory factor 2, *M. musculus*
21841	60,287	11,411	2.40	3.90E − 02	P41827	Heat shock protein 70 B2, *Anopheles albimanus*
86089	269	32	3.08	4.15E − 02	P69309	Polyubiquitin, *Avena fatua*

**Table 3:** Most notable differentially expressed genes (DEGs), comparing HS16 and RC16. HS16 = Heat-Selected clams at 16 °C; RC16 = Random-Control clams at 16 °C. Unless otherwise noted (*), the Transcript ID for each DEG listed in this table contains the prefix “Cluster-17909.” Please see the Supplemental Information for additional information and complete list of DEGs

Transcript ID	Read count (HS16)	Read count (RC16)	Fold change (log2[HS16/RC16])	Adjusted *p* value	Swissprot ID	Swissprot description
105853	63	0	Inf	4.25E − 05	Q6UXZ4	Netrin receptor UNC5D, *Homo sapiens*
121604	53	0	Inf	2.23E − 04	A0JPI9	Leucine-rich repeat-containing protein 74A, *Rattus norvegicus*
*Cluster-35747.4	43	0	Inf	1.97E − 03	P25107	Parathyroid hormone/parathyroid hormone-related peptide receptor, *Didelphis virginiana*
81525	168	12	3.78	2.14E − 03	P20397	Nucleolin, *Xenopus laevis*
29683	45	0	7.39	3.36E − 03	Q96PH1	NADPH oxidase 5, *H. sapiens*
107654	33	0	Inf	1.29E − 02	P15291	Beta-1,4-galactosyltransferase 1, *H. sapiens*
48476	0	262	−Inf	1.21E − 07	G3UYX5	Regulator of G-protein signaling 22, *M. musculus*
45629	0	62	−Inf	4.3E − 05	Q8MJC3	Caspase-3, *Oryctolagus cuniculus*
99251	0	76	−Inf	3.0E − 04	P11586	C-1-tetrahydrofolate synthase, cytoplasmic, *H. sapiens*
45197	0	51	−Inf	3.0E − 04	Q9U8W8	Techylectin-5A, *Tachypleus tridentatus*
129479	0	47	−Inf	4.1E − 04	A6NDX5	Putative zinc finger protein 840, *H. sapiens*
108984	8	121	−3.88	2.14E − 03	Q9D6P8	Calmodulin-like protein 3, *M. musculus*
46678	0	40	−Inf	2.1E − 03	Q6ZV73	FYVE, RhoGEF and PH domain-containing protein 6, *H. sapiens*
101484	0	36	−Inf	4.5E − 03	E1B7L7	Ubinuclein-2, *Bos taurus*
120180	0	39	−7.13	1.12E − 02	P24524	Glycine receptor subunit alpha-3, *R. norvegicus*
90135	3	64	−4.68	2.26E − 02	Q9D3W5	Leucine-rich repeat-containing protein 71, *M. musculus*
121414	0	29	−Inf	2.9E − 02	Q9Z1N9	Protein unc-13 homolog B, *M. musculus*
108364	3	68	−4.34	3.08E − 02	Q8BG94	COMM domain-containing protein 7, *M. musculus*

### Differentially Expressed Genes (DEGs) Induced by Heat Stress

**Fig. 2:**
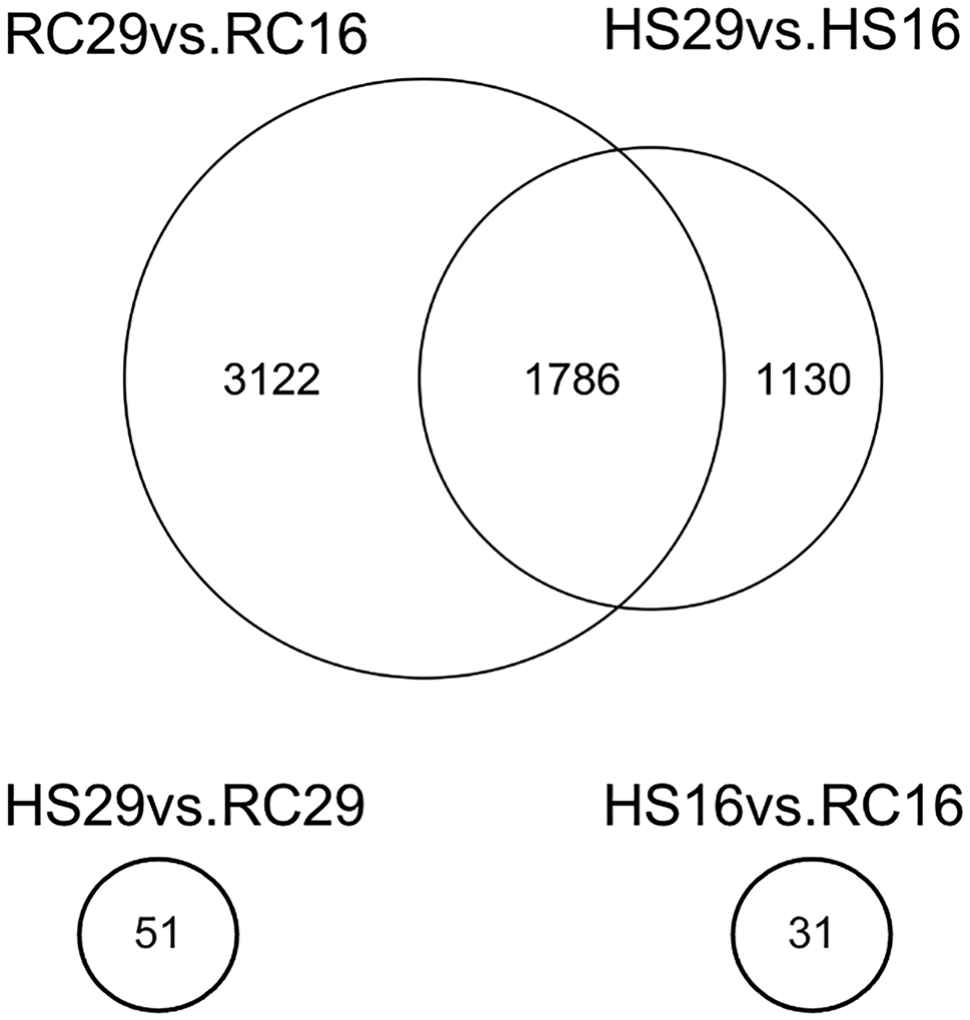
Venn diagram depicting DEGs across group and temperature. (Top) Within-group/across-temperature gene expression. The Venn diagram denotes the number of DEGs identified in each comparison, with the overlapping region showing the number of DEGs shared between the comparisons. (Bottom) Across-group/within-temperature gene expression. Circles denotes the number of DEGs identified in each comparison

**Fig. 3:**
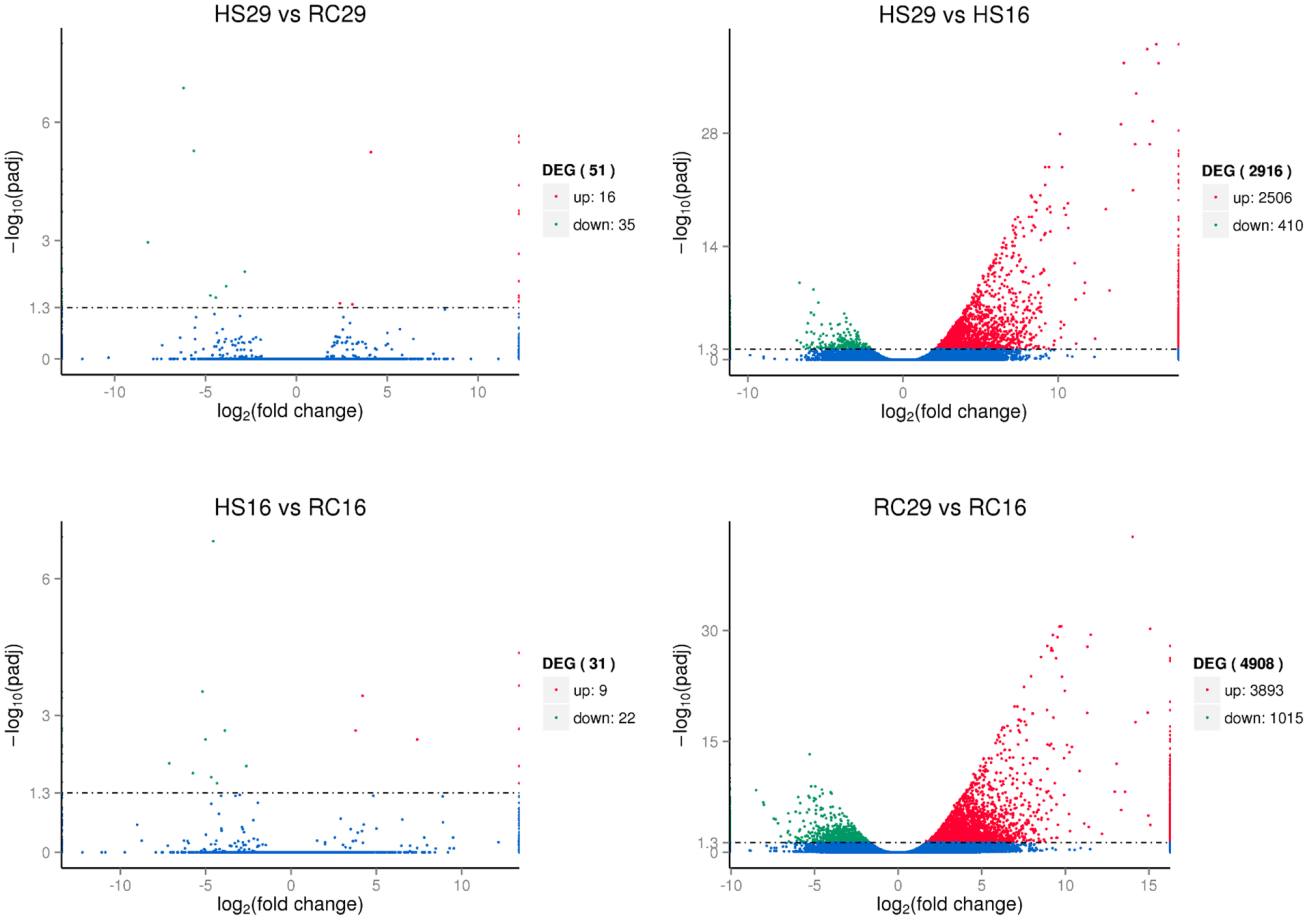
Volcano plots depicting DEGs across group and temperature. Volcano plots show the quantity and statistical significance of up- and downregulated genes for each comparison

The within-group/across-temperature (RC29vs.RC16 and HS29vs.HS16) comparisons were examined to determine how naïve surfclams (RC) respond to heat stress and how it may differ from the ways in which clams that survived a prior lethal heat stress (HS) respond to a repeated exposure. A 6-h heat shock of 29 °C induced a significant response in both the RC and HS groups. The cluster analysis revealed that the RC clams differentially expressed significantly more genes than the HS clams did. Heat stress induced nearly 2000 more DEGs in RC29 compared to HS29. In total, 4908 DEGs were detected in RC29 relative to clams at 16 °C, 79.3% of which were upregulated under heat stress (Figs. 2 and 3). In contrast, 2916 DEGs were detected in HS29 relative to clams at 16 °C and 85.9% were upregulated under heat stress (Figs. 2 and 3). Collectively, 1786 DEGs were shared between the pair of comparisons, and 1638 of those were upregulated at 29 °C (Figs. 2 and 3). The most notable expression patterns were related to genes in the heat shock protein family (HSPs), inhibitors of apoptosis (IAPs), immune-response genes, and oxidative stress-response genes (Table 1).

HS29 and RC29 both exhibited robust differential expression of approximately 60 genes in the HSP family, including *HSP40 (DNAJB)*, *HSP70*, and *Hsp90* (Table 1). Activators and other proteins that interact with HSPs were also identified as DEGs. Compared to their 16 °C counterparts, HS29 exhibited a 75-fold increase in the mean expression of HSPs compared to a 22-fold increase exhibited by RC29.

Expression of genes in the IAP family, such as *Birc2*, *Xiap*, *Diap2*, and *BAK1*, followed a similar pattern (Table 1; Fig. 1). HS29 and RC29 expressed approximately 33 and 47 IAPs, respectively. Although RC29 expressed a greater number of IAPs, the mean expression level of these genes was collectively lower than what was observed in HS29. Whereas HS29 had a 50-fold increase in IAP expression relative to HS16, RC29 had a 19-fold increase relative to RC16. Although RC29 and HS29 did upregulate 15–20 DEGs that promote apoptosis, such as caspase-3, the overall upregulation of pro-apoptosis genes was far lower than the upregulation of IAPs (3- to fivefold increase in mean expression compared to clams at 16 °C).

Numerous immune-related genes, such as toll-like receptors (TLRs), nucleotide-binding oligomerization domain (NOD)-like receptors (NLRs), retinoic acid-inducible (RIG) I-like receptors (RLRs), C-type lectins, and galectins, were also differentially expressed under heat stress (Table 1). In HS29, several TLRs were identified as DEGs, including *TLR4*, *Tlr13*, and *HcToll2*, and every gene was upregulated. Although more TLRs were differentially expressed in RC29 relative to RC16, several were downregulated. Consequently, HS29 had a greater than 20-fold increase in mean TLR expression compared to HS16, while the mean expression of TLRs in RC29 was less than twice as high as RC16. Only one NLR, *nlrc4*, was differentially expressed by RC29, and it was downregulated relative to RC16. HS29 also downregulated this gene relative to HS16, but HS29 also upregulated two other NLRs. Therefore, HS29 had an overall increase in NLR expression. No RLRs were differentially expressed in the HS29vs.HS16 comparison, yet three DEGs in the RC29vs.RC16 comparison had the C-terminal domain of RIG-I. Relative to RC16, RC29 upregulated two and downregulated one of these DEGs, leading RC to exhibit a general decrease in RLR expression at 29 °C. More C-type lectins and galectins were differentially expressed in the RC29vs.RC16 comparison than the HS29vs.HS16 comparison. For RC clams, expression decreased at 29 °C, with RC29 having approximately one-third of the mean expression levels of RC16. By contrast, there was a greater than twofold increase in mean expression of C-type lectins and galectins in HS29 relative to HS16.

Compared to clams at 16 °C, both groups at 29 °C demonstrated significant upregulation of genes that confer protection from oxidative stress. Examples of these genes include superoxide dismutase 1 (*SOD1*), glutathione s-transferase 1 (*GST1*), and peroxidasin (*Pxdn*) (Table 1; Fig. 1). Overall, HS29 differentially expressed approximately 18 antioxidant genes, leading to a 12-fold increase. A similar number of DEGs was identified in the RC29vs.RC16 comparison, but RC29 only exhibited a sixfold increase in mean expression.

Generally, for both groups of clams, the same groups of genes were downregulated at 29 °C relative to 16 °C. Several of genes associated with reproduction were attenuated at 29 °C, including those that code for spermatogenesis associated 6-like protein (*Spata6l*), zygote arrest protein 1 (*ZAR1*), and vitelline membrane outer layer protein 1 (*VMO1*) (Table 1; Fig. 1). Genes associated with the cholinergic nervous system, which play important roles in neural functioning, stress response, and immunomodulation (Shi et al. 2014), were also downregulated in both HS29 and RC29 (Table 1). Specifically, several genes showing similarity to nicotinic and neuronal acetylcholine receptors (e.g., *nAChRβ1*) were downregulated (Table 1; Fig. 1). Notably, half as many differentially expressed acetylcholine receptor genes were identified in the H29vs.HS16 comparison relative to the RC29vs.RC16 comparison (7 vs. 14 DEGs); however, HS29 exhibited a more robust attenuation of these genes compared to RC29 (5.2-fold decrease vs. 1.7-fold decrease in mean expression).

### Differentially Expressed Genes (DEGs) Across Group/Within Temperature

The across-group/within-temperature comparisons (HS29vs.RC29 and HS16vs.RC16) were also examined to determine if HS and RC clams expressed distinct genes under the same conditions. Far fewer DEGs were observed in these comparisons. Only 51 DEGs were identified between RC29 and HS29. Relative to RC29, 35 of those were downregulated and 16 were upregulated in HS29 (Table 2; Figs. 2 and 3). The most notable difference between HS29 and RC29 relates to the expression of a heat shock protein. As mentioned previously, both HS29 and RC29 exhibited robust expression of many heat shock proteins. However, one gene (heat shock protein 70 B2, Transcript ID: 21841) had significantly higher expression (2.4-fold) in HS29 than RC29 (Table 2; Fig. 1). Differences in lipid metabolism gene expression were also apparent. Three lipid metabolism genes were expressed in RC29 but absent in HS29: acetyl-CoA carboxylase (*ACAC*), which is involved with fatty acid biosynthesis, sterol regulatory element-binding protein cleavage-activating protein (*SCAP*), which is involved with cholesterol biosynthesis, and phospholipase (*ABHD3*), which is involved with phospholipid remodeling (Table 2; Fig. 1). HS29 did show significantly higher expression of one lipid metabolism gene, Sn1-specific diacylglycerol lipase beta (*DAGLB*) (Table 2; Fig. 1). Although the expression of IAPs was robust in both RC29 and HS29, both expressed at least one DEG associated with apoptosis induction. Only HS29 expressed mitochondrial apoptosis-inducing factor 1, and only RC29 expressed MAP kinase-activating death domain protein. Other notables DEGs were associated with immunity. Only RC29 clams expressed a probable polyketide synthase (*pks16*), which encodes an enzyme that facilitates the production of antimicrobial agents (Table 2; Fig. 1). Only HS29 clams expressed a gene (Transcript ID: 116707) that shows similarity to a toll-like receptor associated with antimicrobial activity in the triangle-shell pearl mussel *Hyriopsis cumingii* (Ren et al. 2014) (Table 2; Fig. 1).

Only 31 DEGs were identified between RC16 and HS16. Relative to RC16, 22 of those were downregulated and nine were upregulated in HS16 (Table 3; Figs. 2 and 3). HS clams downregulated genes associated with one-carbon metabolism (cytoplasmic C-1-tetrahydrofolate synthase) and innate immunity (techylectin-5A), while upregulating genes associated with chromatin decondensation and ribosome assembly (nucleolin), reactive oxygen species production (NADPH oxidase 5), and protein glycosylation (beta-1,4-galactosyltransferase 1). Both groups expressed genes associated with apoptosis (RC16: caspase 3; HS16: netrin receptor UNC5D) and cell signaling (RC16: regulator of G-protein signaling 22; HS16: parathyroid hormone/parathyroid hormone-related peptide receptor). Both groups also expressed genes that contain leucine-rich repeats (RC16: *Lrcc71*; HS16: *Lrcc74a*).

### KEGG Pathway Enrichment

**Fig. 4:**
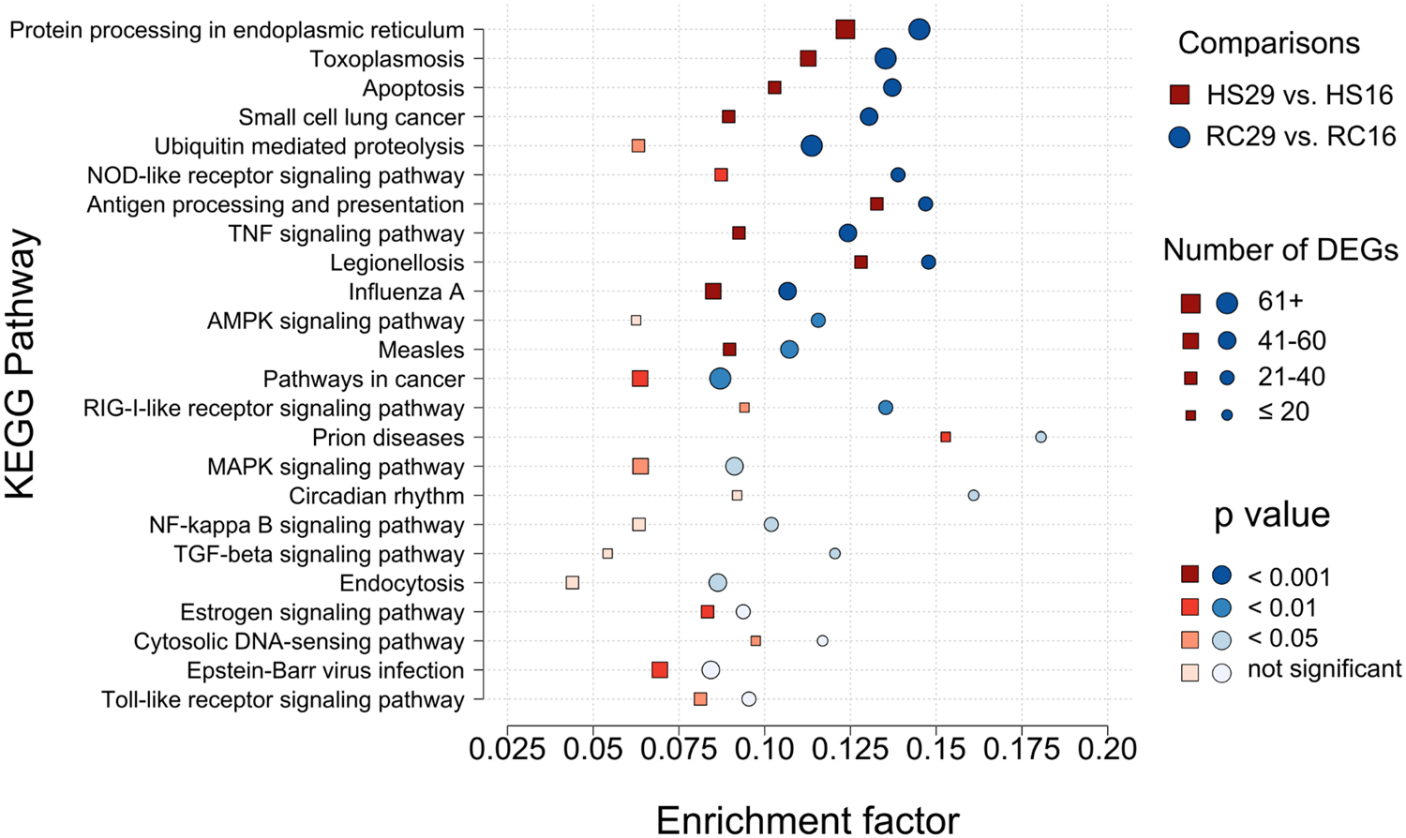
KEGG pathway enrichment for the within-group/across-temperature comparisons. Enrichment factor is the ratio of differentially expressed genes to the background frequency of genes associated with a given pathway. Plotted points convey the comparisons (point shape), the number of DEGs (point size), and the adjusted *p* value (point shade)

Numerous KEGG pathways were significantly enriched with DEGs induced by heat stress. Overall, 20 significantly enriched pathways were identified in the RC group, 19 were identified in the HS group, and 15 pathways were shared across the two groups (Fig. 4). The most significant pathway enriched by both HS29 and RC29, relative to their 16 °C controls, was the “protein processing in endoplasmic reticulum pathway.” This pathway predominantly features proteins of the HSP family, including HSP110, HSP70, HSP90, and DNAJA1 (HSP40), as well as other molecular chaperones that assist in protein folding, sorting, transport, and degradation. Several other enriched pathways also contained HSPs and other chaperones (e.g., calnexin and CDC48). Another pathway that was significantly enriched by both groups at 29 °C was the “antigen processing and presentation pathway.” This pathway contains proteins associated with mounting an immune response (e.g., MyD88) and proteins like serine/threonine kinases (e.g., TBK1 and MAP2K4), which are associated with regulating cell growth and proliferation. Some pathways were significantly inhibited in both groups at 29 °C. These include the “apoptosis pathway” and the “tumor necrosis factor (TNF) signaling pathway.” Both of these pathways are associated with high expression levels of proteins that inhibit programmed cell death (e.g., BCL2, XIAP, BIRC2).

The “toll-like receptor pathway,” which plays a key role in the innate immune system, was only significantly activated by HS29 relative to HS16. This pathway and three others were uniquely significant to the HS group, although they all trended in that direction for the RC group (0.06 < *p* < 0.12, Fig. 4). Conversely, the five pathways unique to the RC group were not enriched to a substantial degree by HS clams (0.15 < *p* < 0.96, Fig. 4). One enriched pathway unique to the RC group was the “AMPK signaling pathway,” which is involved with cellular energy homeostasis. This pathway inhibits protein and fatty acid synthesis while activating fatty acid and glucose oxidation to increase supplies of ATP that may otherwise be depleted during periods of stress. Notably, this pathway was significantly downregulated in RC29 relative to RC16. The “NF-kappa B signaling pathway” was also exclusively significantly enriched by the RC group. Interestingly, RC29 exhibited significant upregulation of both *NFKB1*, a transcription factor that promotes gene expression in response to inflammation and cytokines, and its inhibitor (*cact*), suggesting a complex or conflicted response.

In contrast to the within-group/across-temperature comparisons, few KEGG pathways were significantly enriched in the within-temperature/across-group comparisons. Three KEGG pathways were significantly enriched in RC29 relative to HS29 (*p* < 0.05), and all were related to lipid homeostasis. These include the “fatty acid biosynthesis pathway,” the “aflatoxin biosynthesis pathway,” and “fatty acid metabolism pathway” and were associated with two genes upregulated by RC29, acetyl-CoA carboxylase (*ACAC*) and the probable polyketide synthase (*pks16*). No KEGG pathways were significantly enriched in HS16 relative to RC16.

## Discussion

In this study, the transcriptomes of two populations of surfclams were compared, a control group that was naïve to thermal stress (RC) and a group that was heat-selected for greater heat tolerance (HS) (Acquafredda et al. 2020). The gene expression patterns of these two groups were compared across two temperatures, a stressful condition (29 °C for 6 h) and a favorable or control condition (16 °C for 6 h). Each group/temperature comparison provides insight into the ways in which surfclams cope with heat stress. The RC29vs.RC16 comparison is most representative of the response that wild type surfclams would have during an acute heat stress event. By contrast, the HS29vs.HS16 comparison illustrates the response of a heat-tolerant population that survived a previous exposure of sustained and lethal heat stress. The HS29vs.RC29 comparison is useful because it demonstrates which genes may be most beneficial for surviving heat stress and may facilitate marker-assisted selection. Finally, the HS16vs.RC16 comparison may highlight genes that are constitutively expressed during favorable conditions and predispose the HS clams to higher survival at the onset of heat stress. This would also facilitate marker-assisted selection.

In terms of the total number of genes expressed, RC clams mounted a broader transcriptomic response to 29 °C. RC clams differentially expressed about 1.7 times more genes than HS clams did at this temperature, with approximately 2.8 times more unique DEGs. The expression pattern of RC29 may be interpreted as a broad over-reaction to heat stress, while the response of HS29 is more tempered or focused likely as an adaptive mechanism. This is supported by the fact that while HS29 differentially expressed fewer genes overall, HS29 showed higher expression levels of key stress response genes compared to RC29. Moreover, previous research has shown that bivalves can generate a maladaptive and overactive transcriptomic response when encountering novel stressors. For instance, Ostreid herpesvirus 1 µVar infection causes an explosive oxidative burst in the Pacific oyster (*Crassostrea gigas*), which has been implicated as a contributing cause to the severe mortality of infected oysters (He et al. 2015).

One possible mechanism that explains the response exhibited by HS clams at 29 °C is based on the genetic predisposition of this group. As survivors from a previous heat shock, the HS individuals may be genetically determined to produce a tempered or more focused response whenever they were exposed to heat stress, including during the initial heat challenge selection event (Acquafredda et al. 2020). However, the number of DEGs found in the HS29vs.RC29 and HS16vs.RC16 comparisons was small, indicating one generation of selection did not substantially alter the genome, albeit noticeable differences in heat shock response. An alternative explanation is epigenetic memory. After the initial heat shock event, epigenetic modifications such as DNA methylation or histone modifications (Gavery and Roberts 2010; Wang et al. 2014; Fellous et al. 2015) may have been established in the HS clams, which ensured that gene expression would be moderated or more focused during any subsequent heat stress. Under this hypothesis, the “epigenetic memory” effectively primed the organisms to cope with subsequent stress. If the epigenetic hypothesis is true, our results suggest that the epigenetic memory can last for at least 4 months. Long-lasting epigenetic memory, if confirmed, can be explored to train or condition juveniles for improved performance in the field, providing a new way of developing stress or disease resistant stocks for aquaculture. The two hypotheses may not be mutually exclusive; both mechanisms may contribute to heat response in the preselected clams. Under both hypotheses, the need for HS clams to expend energy on gene expression upon re-exposure to heat stress would be reduced relative to the control clams, further supporting HS clam survival.

Comparisons of both groups across temperatures reveal that heat shock proteins (HSPs) were strongly upregulated at 29 °C. Production of molecular chaperones, like HSPs, during periods of stress is a coping mechanism well conserved among taxa (Sung et al. 2011; Li et al. 2019). HSPs assist with the folding of denatured proteins and newly synthesized proteins during periods of stress (Pockley 2003). In most organisms, constitutive expression of HSPs normally comprises 5–10% of proteins in cells, but this percentage increases dramatically under stress (Pockey 2003). Both RC29 and HS29 clams differentially expressed approximately 60 HSPs, yet it is unclear whether all these DEGs identified in this study represent independent genes as the transcriptome assembly was highly fragmented. Previous research has shown that bivalve genomes are highly polymorphic and contain highly expanded sets of stress and immune response genes such as HSPs, IAPs, and TLRs (Zhang et al. 2012a, b; Guo et al. 2015). Therefore, it is likely that the surfclam genome also contains an abundant array of distinct HSPs. Although a similar number of DEGs were expressed by both groups, the expression of HSPs was thrice as high in HS clams as RC clams. One gene in particular, which most closely resembles *HSP70B2*, was expressed to a significantly greater degree in HS clams than RC clams (*p* = 0.039). Heat stress also induced upregulation of this gene in the Pacific oyster and the northern quahog (*Mercenaria mercenaria*) (Valenzuela-Castillo et al. 2015; Song et al. 2022). Together, these results imply that robust expression of HSPs is foundational to enhanced heat tolerance in surfclams.

Both groups expressed genes that facilitate apoptosis at 16 °C, which likely reflects the fact that apoptosis is a process that occurs as a part of an organism’s normal growth and maintenance. Yet programmed cell death is also a consequence of temperature stress (Menike et al. 2014). While both groups expressed genes that promote apoptosis at 29 °C, the induction of apoptosis was likely largely mitigated because both HS and RC clams significantly upregulated between 30 and 50 inhibitors of apoptosis (IAPs). Significant upregulation of an expanded set of IAPs is a key feature of stress (Zhang et al. 2012a, b; Guo et al. 2015; Song et al. 2021) and immune (He et al. 2015; Chan et al. 2021; Witkop et al. 2022) responses in bivalve mollusks. While the RC group expressed more IAPs, the HS group exhibited a stronger response; the HS group exhibiting a 50-fold increase in IAP expression, while the RC group had only a 19-fold increase. These results suggest that robust expression of IAPs is of paramount importance to surfclam heat tolerance.

The results of this study also indicate that oxidative stress response is another important component of surfclam heat tolerance. Both preselected and control groups highly expressed genes that mitigate the effects of reactive oxygen species (ROS), which often co-occur with apoptosis during periods of stress. In all cells, small amounts of ROS occur as byproduct from normal oxidative metabolism (Simon et al. 2000). However, periods of stress can lead to immense increases in ROS production, destroying numerous cellular components and triggering apoptosis (Simon et al. 2000). In other bivalves, genes that help quench ROS are among those most highly upregulated during thermal stress (Meistertzheim et al. 2007; Truebano et al. 2010; Menike et al. 2014). Although a similar number of DEGs were expressed by RC29 and HS29, the latter demonstrated stronger expression of oxidative stress response genes. Furthermore, HS29 showed a stronger attenuation of nicotinic acetylcholine receptors. Genes encoding nicotinic acetylcholine receptors are greatly expanded in bivalve mollusks probably as a compensation for reduced nervous systems for coping with dynamic stressors under stationary life (Jiao et al. 2019). In the Zhikong scallop (*Chlamys farreri*), acetylcholine inhibited oxidative stress response genes such as superoxide dismutase after immune system stimulation (Shi et al. 2014). If the surfclam cholinergic nervous system functions similarly, these results may point to an additional mechanism used by surfclams to bolster their oxidative stress response, whereby genes involved in the innate immune response also respond to heat stress.

Interestingly, HS and RC clams had notably different expression patterns of immune-related genes in response to heat stress. Compared with humans, the fruit fly *Drosophila melanogaster*, and other model organisms, bivalves have an expanded set of innate immune receptor genes, such as toll-like receptors (TLRs), retinoic acid-inducible (RIG) I-like receptors (RLRs), and C-type lectins (Zhang et al. 2015). These expanded gene families have supported structural and functional diversification, causing many immune-related genes to adopt new roles, particularly with respect to abiotic stress response (Guo et al. 2015; Guo and Ford 2016). More than half of all immune-related receptors and adaptors identified in the Pacific oyster are associated with abiotic stress response (Zhang et al. 2015). Specifically, members of the TLR family, which generally respond to pathogenic microbes and stimulate inflammatory signaling cascades, are also expressed when bivalves experience temperature and other abiotic stress (Kawaski and Kawai 2014; Zhang et al. 2015; Huang an Ren 2019). Likewise, RLRs, MyD88, and TNF-related genes are also upregulated during temperature stress (Zhang et al. 2015). These genes may be upregulated to defend against infections that often co-occur with heat stress, or they may have developed other stress-mitigating functions (Guo et al. 2015). In this study, HS29 exhibited a general upregulation of TLRs, NLRs, genes for carbohydrate-binding proteins (i.e., C-type lectins and galectins), and other immune-response related genes and pathways. By contrast, RC29 had a more mixed response. RC29 did uniquely express a probable polyketide synthase, which indicates this group may have been producing antimicrobial molecules (Sabatini et al. 2018). However, RC29 generally downregulated or had relatively low expression levels of members of the most prominent immune-response gene families. For instance, TLRs were highly upregulated by HS clams under stress (20-fold increase compared to clams at 16 °C), and at least one DEG was both significantly upregulated and unique to this group. Many TLRs were downregulated in RC29, and the overall expression of TLRs was less than twice as high as RC16. Additionally, while C-type lectins and galectins were generally upregulated in HS29, RC29 showed the opposite trend. Together, these results suggest that regulation of certain homologs of immune-related genes may be key components of enhanced surfclam heat tolerance, and these genes may be good candidates for selection by breeding programs. However, additional research is required to determine the precise roles that these genes play in mitigating heat stress.

Differences in lipid metabolism gene expression were also observed. At 29 °C, the RC clams upregulated genes and pathways associated with fatty acid synthesis, repair, and remodeling, whereas the HS clams did not. This may reflect subtle differences in how HS and RC clams responded to heat stress. The membranes of polar or cold-adapted animals tend to have more unsaturated fatty acids comprising the phospholipids of their cell membranes; in contrast, animals in temperate or tropical climates tend to have more saturated acids, like cholesterol, contributing to their cell membranes (Palmerini et al. 2009). When some bivalve species suffer heat stress, their lipid content becomes reduced, either because their lipids are metabolized as an energy source (Anacleto et al. 2014), or they undergo the process of lipid peroxidation, where lipids are degraded by ROS (Abele and Puntarulo 2004). Surfclams are also known to exhibit a homeoviscous adaptation response to seasonal changes in temperature, adjusting membrane fluidity by altering the fraction of saturated and polyunsaturated fatty acids that comprise the phospholipids of their cell and mitochondrial membranes (Munro and Blier 2015). Moreover, membrane remodeling is one hypothesis put forth to explain how multiple oyster species have induced thermal tolerance after being pre-treated with a short-term heat shock (Shamseldin et al. 1997; Zhang et al. 2012a, b; Periera et al. 2020). We hypothesize that the HS clams may have remodeled their membranes after surviving their initial heat shock, thus predisposing them to cope with the stressful conditions of this experiment. However, future studies should further examine whether membrane remodeling plays any role in surfclam heat tolerance.

One well understood physiological consequence of heat stress in surfclams is a reduced scope for growth, which includes the energy available for reproduction (Munroe et al. 2013; Narváez et al. 2015; Hofmann et al. 2018). Prolonged exposure to unfavorable temperatures can limit reproductive success (Munroe et al. 2013). If the stress continues, scope for growth can become negative, and under those conditions, starving clams may reabsorb their gonads as an energy source (Kim and Powell 2004). Temperature stress has also been associated with abnormal gonad development in surfclam populations at the southern edge of their range (Kim and Powell 2004) as well as in near shore areas of New York (Hornstein et al. 2018). In this study, HS29 and RC29 clams downregulated genes associated with the production of sperm and eggs (e.g., *Spata6l* and *VMO1*) and genes involved in the oocyte/embryo transition (e.g., *ZAR1*). These results provide supporting genetic data to the link between heat stress and reduced gametogenic functioning. It also demonstrates that these impacts can occur as soon as 6 h into a heat shock. This information may be particularly salient for growers who may be holding or ripening broodstock on shallow coastal farms that experience temperature fluctuations on tidal and diel scales.

Ocean warming presents numerous challenges for marine species. For largely sessile organisms like infaunal bivalves, persistence depends on the capacity of populations to adapt or evolve to these rapid environmental changes (Bitter et al. 2019). The present study elucidates the gene expression profiles of a surfclam population that previously survived a lethal heat challenge, while also comparing those patterns to clams that are more representative of individuals found in the wild. Based on the findings of this study, when surfclams undergo acute heat stress, they respond by robustly expressing HSPs, IAPs, and genes mitigating ROS production. Although HS clams expressed fewer genes overall during heat stress, HS clams had a more focused response, exhibiting stronger expression of key stress-response gene compared to RC clams. This work should inform future breeding programs that attempt to breed surfclam for greater heat tolerance via marker-assisted selection, yet more work is required to fully understand the surfclam’s adaptive capacity to thermal stress. Proteomics research should be conducted to elucidate which of the identified heat-induced transcripts are ultimately translated and put into action by surfclam cells. Confirming epigenetic memory in heat response would provide guidance on future directions of stock improvement. Additionally, more research should be devoted to understanding whether there are trade-offs associated with enhanced heat tolerance and understanding how chronic exposure influences surfclam gene and protein expression. In the Pacific oyster, many of the genes that were upregulated during an acute heat challenge were not significantly expressed during chronic exposure, implying that oysters respond to short-term and long-term thermal stresses using different mechanisms (Clark et al. 2013). Since surfclams are more likely to experience long-term stress both on farms and in the wild, the surfclam’s response to prolonged thermal stress should be examined in future studies. Finally, future research efforts should aim to elucidate the standing genetic variation among surfclam individuals and populations to determine whether wild surfclams throughout their geographic range have the innate adaptive capacity to persist in a warming ocean.

### Supplementary Information

Below is the link to the electronic supplementary material.

Supplementary file1 (XLSX 4101 KB)

Supplementary file2 (XLSX 345 KB)

Supplementary file3 (PDF 290 KB)

## Data Availability

The datasets generated during and/or analyzed during the current study are hosted at the Rutgers University Haskin Shellfish Research Laboratory (Port Norris, NJ), and portions can be provided from the corresponding author on reasonable request. Raw sequence data were submitted to the National Center for Biotechnology Information (NCBI) Sequence Read Archive (SRA) under BioProject ID #PRJNA596792. The data can be accessed at https://www.ncbi.nlm.nih.gov/sra using the biosample numbers: SAMN13638038–SAMN13638049. The Transcriptome Shotgun Assembly (TSA) project has been deposited at DDBJ/EMBL/GenBank and can be accessed at https://submit.ncbi.nlm.nih.gov/about/tsa/ using the accession number: GKQA00000000.
