# Supplementary figures and images for: Transcriptomic Response of the Atlantic Surfclam (Spisula solidissima) to Acute Heat Stress
Source: Mar Biotechnol (NY). 2024 Jan 19;26(1):149–68. doi: 10.1007/s10126-024-10285-0 (PMC10869415; doi:10.1007/s10126-024-10285-0)

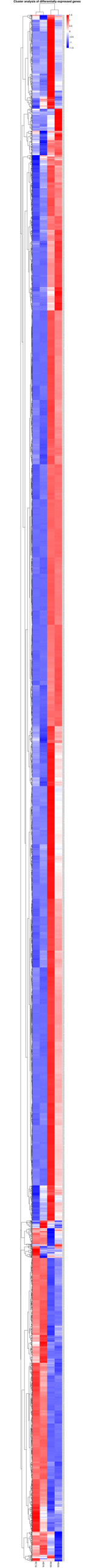

Supplement: Supplementary file 3 — Supplementary file3 (PDF 290 KB) [file 10126_2024_10285_MOESM3_ESM.pdf]
